# Ethical issues in oncology practice: a qualitative study of stakeholders’ experiences and expectations

**DOI:** 10.1186/s12910-022-00803-x

**Published:** 2022-06-30

**Authors:** Chiara Crico, Virginia Sanchini, Paolo G. Casali, Gabriella Pravettoni

**Affiliations:** 1 Department of Oncology and Hemato-Oncology, University of Milan, Milan, Italy; 2 Applied Research Division for Cognitive and Psychological Science, European Institute of Oncology IRCCS, Milan, Italy; 3 Fondazione IRCCS Istituto Nazionale Tumori, Milan, Italy; 4 Department of Public Health and Primary Care, Centre for Biomedical Ethics and Law, KU Leuven, Leuven, Belgium

**Keywords:** Clinical ethics support, Ethical issue(s), Semi-structured interviews, End-of-life, Medical communication, Resources allocation, Clinical Ethics Committee

## Abstract

**Background:**

Clinical Ethics Support Services (CESS) have been established to support healthcare professionals in addressing ethically sensitive issues in clinical practice and, in many countries, they are under development. In the context of growing CESS, exploring how healthcare professionals experience and address clinical ethics issues in their daily practice represents a fundamental step to understand their potential needs. This is even more relevant in the context of extremely sensitive diseases, such as cancer. On this basis, we carried out a qualitative study conducting in-depth semi-structured interviews with stakeholders of a major comprehensive cancer centre in Italy, with the twofold aim of investigating what ethical issues arise in the context of clinical oncology and how they are addressed, as well as stakeholders’ expectations about a potential CESS to be implemented within the Institution.

**Methods:**

The study was conducted within the theoretical framework of Grounded Theory. Participants were healthcare professionals and other key stakeholders working within the cancer centre. The semi-structured interview aimed at exploring common ethical aspects of oncology, investigating stakeholders’ professional experience in dealing with clinical ethics issues, their expectations and requests regarding ethics support services. Transcripts of the interviews were coded and analysed according to the principles of Grounded Theory.

**Results:**

Twenty-one stakeholders were interviewed. Our analysis showed a wide consensus on the identification of ethically relevant issues, above all those concerning communication, end-of-life, and resource allocation. The absence of institutional tools or strategies to address and manage ethical issues at the patient bedside emerged, and this is reflected in the widespread request for their development in the future. The ideal support service should be fast and flexible in order to adapt to different needs and clinical cases.

**Conclusions:**

The interviewees showed a limited degree of ‘ethical awareness’: despite having reported many issues in clinical practice, they could hardly identify and describe the ethical aspects, while  complaining about a lack of ethical resources in their management. To build a truly effective support service, it therefore seems appropriate to take such context into consideration and address the emerged needs. Ethical sensitivity seems to be key and it becomes even more relevant in critical clinical areas, such as the therapeutic pathways of terminally ill patients.

**Supplementary Information:**

The online version contains supplementary material available at 10.1186/s12910-022-00803-x.

## Background

A greater awareness about ethical implications of clinical decision-making, together with the advances in biomedical research and technology, led to radical changes in the health care domain. These changes brought healthcare professionals to face ethical challenges and ethically sensitive issues related to clinical practice [1], whose management often requires dedicated clinical ethics expertise.

Clinical Ethics Support Services (CESS) have been developed to address these issues: supporting healthcare professionals, patients, and their families facing ethical concerns in clinical practice [2], and guiding the clinical decision-making process while protecting patients’ rights [2, 3].

CESS may be provided in different ways, depending on numerous variables such as the social and cultural context, and the availability of resources [4]. However, three are now considered the standard institutional forms to provide CESS: Hospital or Clinical Ethics Committees (CECs), generally composed of a heterogeneous and multidisciplinary team of professionals; Ethics Consultants, either working individually or in small teams to help healthcare staff identifying, analysing and proposing resolutions to clinical ethics issues; Moral Case Deliberation (MCD), which is a method whereby a group of professionals systematically reflect on precise moral questions concerning concrete clinical cases drawn from clinical practice [2, 4–6]. CECs are currently the most widespread form of CESS, although their very notion can vary greatly from country to country [4, 7].

While CESS have become widespread in North America as early as in the 1980s, they began to develop in Europe only a decade later, at a slow pace and not in all countries. Only recently CESS have started to develop extensively in most European countries, although with considerable differences [8, 9]. For instance, some countries enforce the establishment of CESS by law, while others do not provide any regulation at all [4]. Italy belongs to the latter, as it still does not have a normative framework for CESS [10]. Italian law makes no distinction between the different types of ethics committees. Therefore, ethics committees are required to deal with research-related activities, but they are also formally in charge of the tasks that the literature attributes to CECs (i.e., case consultation, ethics training of health care professionals, and ethics policy revision) [11, 12]. However, most ethics committees are overburdened with research-related work and do not have the time/resources to perform other activities, therefore operating only as Research Ethics Committees (RECs) [13–15]. Trying to address this issue and ensuring that the ethical dimension of care is given adequate attention in healthcare facilities, the Italian National Bioethics Committee (INBC) has urged the government to separate the two different types of committees by establishing CECs alongside the current RECs, assigning to CECs all those activities mentioned above relating to clinical practice [16, 17]. To date, the INBC’s request has not been yet met nation-wide, but a few Regions have taken actions to fill this gap by establishing CECs [16].

In line with this trend, the Fondazione IRCCS Istituto Nazionale dei Tumori, a comprehensive cancer centre based in Milan, Italy, whose ethics committee is almost fully committed to the ethics review of clinical trials, decided to devise a CEC devoted to support its staff in dealing with ethical issues in clinical practice. In line with this purpose, understanding the experiences of the stakeholders working within this cancer centre with ethically sensitive issues in clinical practice is crucial to outline their needs for support; this is a fundamental preliminary operation to design a body tailored to the real specific demands of healthcare professionals and patients [18]. This study is a consistent part of the PhD project of the first author (CC).

Moreover, being the Fondazione IRCCS Istituto Nazionale dei Tumori exclusively devoted to cancer care, we expect mostly oncology-related ethical issues to arise. Since cancer has a peculiar profile, different from other chronic non-communicable diseases [19], it is equally important to bear in mind its peculiarities  also from the ethical standpoint. The cancer patient’s pathway is in fact marked by specific phases of disease, which may pose peculiar ethical problems—such as those entailed by major surgery or toxic medical therapies as well as issues like heredofamilial cancer risk or end of life. The psychological impact that a cancer diagnosis implies [20, 21] is also a well-known and important aspect to be considered in the care pathway.

Based on these premises and with the overarching aim of establishing a CEC at the Fondazione IRCCS Istituto Nazionale dei Tumori, we carried out a qualitative study with a twofold purpose. First, the study was directed at understanding how stakeholders working within this institution experience ethical issues: namely, what kind of ethical issues they encounter in their daily lives and what strategies they use to deal with them. Secondly, we wanted to explore their expectations about a potential CEC. The stakeholders interviewed were key professional figures working within the Institution, such as healthcare professionals (e.g., physicians and nurses), REC’ members and Patient Advocacy Group’ members.

As there are few studies in the literature with similar aims, most of which using surveys [22–24], we adopted a qualitative approach, as this allowed us to deepen the participants’ perspectives while also enabling some space for interpretations [25, 26]. Semi-structured interviews were particularly suited for our goals, as they allow an in-depth investigation, providing the opportunity to delve into the experiences and perspectives of stakeholders on clinical ethics issues [27]. 

Drawing from these investigations, we expected to gather useful information that will contribute to the setting up of the CEC and to its proper functioning within the Institution.

## Methods

The methodology whereby this study was designed and conducted is reported following the items in the COREQ checklist [28], attached with the manuscript supplementary materials.

### Study design and population

A devoted research protocol for the qualitative study was designed by the first and second author (CC and VS), revised and approved by the third author (PGC), Principal Investigator (PI) of the study. The research protocol was submitted in February 2019 to the REC of the Fondazione IRCCS Istituto Nazionale dei Tumori (Research Protocol Title: “Investigation of ethical-clinical expectations within an oncological institution”; Code “INT 65/19”), and approved in March 2019.

About the inclusion criteria for participants’ enrolment, we invited a sample as inclusive and unbiased as possible, involving professionals from several areas from the Institution. In particular, in order to elicit a multidisciplinary and inclusive participation, the Directors of the clinical units of the Institution were invited to participate personally or to indicate someone from their team willing to take part to our research. Moreover, in order to have patient representation, we invited the members of the Patient Advocacy Groups operating within the Institute. Lastly, since the REC of the Fondazione is theoretically in charge of all the activities that the future CEC will carry out, we also invited all its members to participate in the study.

The enrolment phase lasted from June to December 2019. In June, an email was sent by the third author and PI of the Study (PGC) to the selected group of stakeholders of the Institution (100 people in total). After the first email, follow-up e-mails were sent to solicit participation to those who did not reply to the first email. Given the voluntary nature of participation in our study, after three unanswered emails, we no longer solicited participants, respecting their tacit decision not to take part in our research.

The order of the interviewees, present in Table 1, followed the chronological order of sampling. Following the logic of “theoretical sampling” [29], we progressively recruited research participants according to the first emerging results.

**Table 1:** Demographic characteristics and professional information of the study sample

No.	Age	Profession	Institute Department (if applicable)	REC Member	Professional experience (years)
1	45–60	Medical Oncologist	Genitourinary Medical Oncology	No	> 10
2	45–60	Anaesthesiologist	Intensive care Unit	No	> 10
3	45–60	Medical Oncologist	Paediatric Oncology	No	> 10
4	45–60	Medical Oncologist	Paediatric Oncology	No	> 10
5	45–60	Clinical Psychologist	Senology	No	> 10
6	Over 60	Radiation Therapist	Prostate Cancer Unit	No	> 10
7	30–45	Geriatrician	Patient support care	No	> 5, < 10
8	30–45	Medical Oncologist	Soft Tissue Sarcomas	No	> 5, < 10
9	30–45	Medical Oncologist	Hemato-Oncology,	No	> 10
10	45–60	Surgeon	Soft Tissue Sarcomas	No	> 10
11	45–60	Patient Advocacy Group member	/	No	> 10
12	Over 60	Medical Oncologist and Nuclear Physician	/	Yes	> 10
13	45–60	Medical Oncologist and Palliative Care	/	Yes	> 10
14	Over 60	Attorney at law	/	Yes	> 10
15	45–60	Case manager	Soft Tissue Sarcomas	No	> 10
16	Over 60	Paediatrician Hemato-oncologist	/	Yes	> 10
17	Over 60	Medical Oncologist	/	Yes	> 10
18	Over 60	Patient Advocacy Group member	/	Yes	> 10
19	45–60	Head nurse	Hospice	Yes	> 10
20	30–45	Research Nurse	Head and neck oncology	No	> 5, < 10
21	45–60	Chaplain	/	Yes	> 10

The emails sent to prospective interviewees contained a brief explanation of the study primary and secondary objectives, along with the invitation to express suggestions related to the study, as well as the request for availability to undergo the interview. In order to allow spontaneous and—as much as possible—unbiased answers, participants were given minimum information in advance about the topics of the interview. Those who responded positively to the invitation were then contacted by the first author (CC) via email or telephone to arrange a meeting for the interview.

### Data collection

Data were collected through in-depth semi-structured interviews and a demographic questionnaire (Table 1). In most cases, the interviews took place at the Fondazione IRCCS Istituto Nazionale dei Tumori, with no third parties assisting. The first author (CC), who had no prior relationship with the participants, conducted the interviews, audio recording them and taking field notes and memos to register non-verbal elements of communication. The interviewer provided prompts, clarifications, and examples as needed to better clear up any doubts about the interview questions. She then transcribed verbatim the audio files and then translated them in English for further analysis. Transcripts were sent to participants upon request but no corrections, comments or notes were made to the texts. Data saturation was reached after twenty-one interviews, once we considered to have interviewed a sufficiently varied sample of relevant stakeholders, representing key figures from the Institution, while also having obtained sufficiently content-rich data material.

### Interview scope and questions

The in-depth interviews followed a semi-structured outline based on ten questions (the complete set of questions is present in Additional file 1: Appendix 1) on the following issues: common ethical problems in oncology, from the point of view of both clinicians and patients; individual and institutional strategies currently implemented to address them; wishes and expectations for a potential clinical ethics support service.

The interviews’ topics and questions have been formulated considering both the context of the oncological institution where the study was carried out, and the data available in the literature, referring to qualitative studies with similar purposes and characteristics [30, 31].

Given the nature of the study, focused on bioethical issues in clinical oncology, the interview script was developed by the first author (CC), PhD student in Medical Humanities, with the support and review of the second author (VS), PhD and senior bioethicist, and the third author (PGC), medical oncologist. The interview script was pilot checked by a small group of three people with different expertise in order to verify the comprehensibility of the questions. The script was also attached to the trial protocol submitted to the REC, which did not recommend any modification.

Despite the guided structure, the in-depth interviews followed the flow of the conversation and the questions were adapted from time to time to the interviewee’s sensitivities and interests, without constraining the conversation on specific topics [26, 32]. In general, the questions aimed at eliciting stakeholders’ personal experiences with ethically challenging situations encountered in their clinical daily practice: whether they faced some ethically sensitive issues, in what clinical domains, their content; whether they asked for any support, and to whom, and whether they were satisfied with the received support. In addition to the experiences, our questions aimed also at investigating stakeholders’ expectations towards the potential implementation of a CESS in the Institution: whether they would consider it valuable, which kind of ethical issues should be addressed first, how the service should be structured, etc.

### Data analysis

To ensure the internal reliability of the analyses, the entire process was performed by two coders, the first and second author (CC and VS). Like the first author, the second author also had no prior acquaintance with the participants and no expectations regarding the context in which interviews were conducted. Both the authors (CC and VS) listened to the audio recordings, read the transcripts and analysed the transcriptions individually, following the criteria of Grounded Theory [25, 26, 29, 33, 34]. In the first phase (open coding), the first and second author coded the transcripts line-by-line, assigning a code to each emerging theme that was faithful and descriptive of the subjects' experience, attempting to bring out as many concepts as possible. In the second phase of analysis (axial coding), the same two authors (CC and VS) gradually identified the common aspects among the subjects’ transcript responses and built broader categories accordingly. In this phase, it was often necessary to analyse and clarify the terms used by the participants (different participants often used different and rarely standard terms). In the last phase of analysis (selective coding), the relationships between the conceptual categories were outlined, thus leading to greater abstraction from the empirical data and the identification of the core categories, on which our interpretative model is based.

The first author (CC) used the software Nvivo 12 to conduct the analysis, while the second author (VS) performed coding by hand. To increase reliability, after the two authors performed the analysis individually, they presented each other the codes for comparison and mutual evaluation [35]. Codes and categories that were not agreed upon were debated and discussed between the first and second author (CC and VS) until consensus was reached. The codes and themes that emerged were then discussed with the third author (PGC), a medical oncologist working within the Institution, to discuss the validity of the interpretation of the texts given by the first two authors.

## Results

### Description of study participants

Of the hundred people from the Fondazione IRCCS Istituto Tumori Milano invited to participate in the study, forty-one people expressed their wish to attend the semi-structured interviews, fifty did not give explicit availability to the interviews but they appreciated and welcomed the study proposal, nine never replied.

Among the forty-one people who were willing to participate to the semi-structured interviews, twenty-one were actually available and were enrolled in the study; the remaining twenty potential candidates eventually declined their participation, mainly referring to a lack of time as justification. The twenty-one participants included belonged to one or more of the following categories: eight REC’s members,^1^ six head of departments, three staff physicians, two members of Patient Advocacy Groups, two nurses, one medical director, one case manager. For the demographic details of the study participants, see Table 1. The interviews were conducted by the first author (CC) and lasted from 15 to 76 min, with an average of 42 min. Healthcare professionals (physicians, surgeons or nurses) staff of the Institution who participated in the study belong to one of the following units: Mesenchymal Soft Tissue Tumours (sarcomas) Oncology (N = 3), Clinical Psychology (N = 1), Genitourinary Oncology (N = 1), Intensive Care (N = 1), Paediatric Oncology (N = 2), Patient Support Treatment (N = 1), Haematology (N = 1), Oncological paediatrics (N = 2), Radiotherapy (N = 1), Palliative Care, Pain Therapy and CPR (N = 1). Members of the REC of the Institution enrolled in the study had clinical experience in Palliative Care (N = 3), Diagnostic Imaging and Radiotherapy (N = 1), Paediatrics Oncology (N = 1). The remaining participants among the REC members are a Patient Advocate, an attorney, the hospital chaplain. Two of the interviewed members of the REC were also part of the hospital staff.

### Emerging clinical ethics themes

About half (6/10) of the guiding questions in the semi-structured interview (see Additional file 1: Appendix 1) aimed at investigating the experiences of healthcare staff and other relevant stakeholders working within the Fondazione IRCCS Istituto Nazionale dei Tumori, in addressing ethical issues occurring in clinical practice. Such questions were firstly purposed to understand what issues are perceived by participants as ethically controversial and whether they were able to recognize all the ethical aspects of the problems they face in their clinical daily practice. Furthermore, the questions were also intended to understand whether and what clinical ethics issues are reported more frequently than others, and how they are addressed and/or solved by stakeholders.

Based on the collected answers, we first observed that, when asked about clinical ethical issues, over half of interviewees (11/21) could not define what a clinical ethics issue is, needed some examples to understand the concept, or reported purely clinical or psychological issues. However, in all the interviews critical issues emerged, although not always qualified as such by the participants. Thus it was possible for us to uncover the underlying ethical concerns, to infer the themes through coding and identify the relationships between the themes. This process was carried out by the first (CC) and the second author (VS). On the grounds of the described analysis, we listed the most frequent clinical ethics issues, as reported by the participants (see Table 2).

**Table 2:** Description of the themes emerged through the coding process and their sub-categories

Themes	Description	Examples of potential issues
Communication issues	It includes all the issues related to the content of communication as well as to the process of communication	Communicate the worsening of the prognosis
		Lack of empathy
		Transparency and completeness of information
		Unreliable or non-filtered information
		Incomprehension among colleagues
		Presence of potential barriers [language, low health literacy]
End-of-life	It includes all those controversial issues related to treatment in the terminal phase of oncological disease, mainly from a moral but also legal and regulatory standpoint	Assisted suicide
		Advance Directives
		Palliative deep sedation
		Withholding/withdrawing treatment
		Transition from active therapies to palliative care
		Feeling of abandonment of terminally ills
Resource allocation	It refers to obstacles to a fair distribution of healthcare resources; in this study, resources are intended primarily as clinical and surgical time, availability of drugs and treatments, and accessibility to updated therapies	Economic discrimination [high cost of branded drugs, new drugs available only for purchase]
		Territorial differences in therapies availability
		Age-based restriction of therapeutic proposals (Ageism)
Genetic mutations: testing and counselling	It refers to innovative genetic testing techniques open up a wide range of scenarios, all of which raise ethical issues. This category is at the crossroads between the issues of decision-making, informed consent, privacy and patient autonomy	Communication of the result of the genetic test to relatives
		Understanding the meaning of genetic testing
		Awareness on therapeutic choices
		Prognosis reliability
Informed consent	It refers to problems related to the principle of self-determination and the right to information, such as patients failing to understand clinical information, due to the lack of health literacy, awareness of treatment options, due to their diminished autonomy (i.e. minors and adults with significant cognitive impairment)	Informed Consent in paediatrics
		Right to information
		Patient manipulation towards selected therapeutic choices
Medical Culture	It refers to cultural aspects of medical practice with potential ethically relevant impacts on the former. It includes the contemporary tendency to conceive the medical act as a procedural activity and physicians as mere technicians	“Acting” medicine vs “thinking” medicine
		Concept of death and mortality
		Terminal illness as failure
		Cancer as taboo word
Medical Decision Making	It includes all borderline cases in which standard therapeutic guidelines and protocols cannot simply be top down applied, or conflict with the patient’s values (i. e. in the absence of sufficient scientific evidence to support a specific therapeutic choice or in cases of uncertain prognosis)	Uncertainty of prognosis in rare cancers
		Newly diagnosed cancer in the elderly
		Cancer during pregnancy
		Jehovah witnesses and surgery
Practical problems	It includes issues that are neither purely clinical nor purely ethical, but which are perceived as ethically worthy since they affect the quality of care, albeit indirectly	Obsolescence of office supplies
		Limited medical time

Table 2 reports the themes categorized from the most to the less recurrent ones: communication issues, end-of-life issues, medical-decision-making, genetic testing and counselling, resources allocation, informed consent, privacy issues, issues related to the “medical culture”, and practical problems. In order to determine the frequency of the themes, we assigned a numerical value to the recurrences with which they were mentioned by the participants. It should be noted that not in all cases the number reflects the importance assigned by each participant to the single theme—it may be that an issue (and related theme) was mentioned briefly by an interviewee and thoroughly elaborated by another one: the order of the themes provides a preliminary view on the topic, based on how often an issue was mentioned.

#### Communication issues

In the next sections, we will explain in greater details some of the issues and problems listed in Table 2, which will be then further addressed in the Discussion section.

With respect to the first theme, “Communication”, almost all interviewees reported that physicians struggle to communicate bad news to patients and/or family members. According to certain categories of participants—in particular nurses and patient representatives, but also physicians from critical clinical areas (n. 2, 5, 11, 19, 20)—doctors often fail to clearly explain what is going on, tend to express in vague terms situations such as the worsening prognosis and the implications arising from it (e.g. the nearing of death, end-of-life options such as the DNR order, when to start palliative care, to name a few). According to some, these struggles have to do not only with the inherent difficulties of communicating bad news, but also with a sense of defeat towards the disease:

But the oncologist has, even rightly, the goal of treating the patient, so after 3 years in here I somehow realize that for them it's almost like a defeat to let the patient go [Interviewee n. 7].

(…) the doctor himself, as well as the nurse, never gives up in the face of illness [Interviewee n. 20]

Other reported problems relate to transparency and/or completeness of communication, with information being conveyed in such a way as to direct the patient towards some treatment choices rather than others. An example cited several times by our sample is the case of prostate cancer: in some cases, patients with early-stage prostate cancer have multiple and equally valid treatment options. In this case, the doctor’s role in informing the patient about potential treatment options plays a crucial role in guiding the patient towards the most suitable choice for them. However, patients do not always receive adequate information about their possible therapeutic path and may be guided to one or the other by means of poor information:

I experience, let's say, ethical issues for example in patients I receive with prostate cancer, who have been informed by a fellow surgeon, who did not fully inform them of their treatment options, or worse, who denigrate for instance an equally effective therapy or, let's say, who put other personal interests at the centre, instead of patients. In other words, they use patients as means and not as ends. [Interviewee n. 6].

Participants also highlighted several problems related to the communication process. Many interviewees, from both the healthcare staff and patient advocacy groups, report a lack of empathy among physicians (n. 1, 5, 6, 10, 11, 18). Participants perceive this lack as a significant issue, especially if a recovery is no longer possible.

Obviously in a context in which, in fact, your healing power is relative - and it's not zero, far from it, meaning that anyway cancers heal, it's not that they don't heal, but anyway they don't heal all - all the other aspects of the relationship become relevant too. They would be relevant anyway, but all the more so where you are not able to guarantee everyone the success of what you are doing, in short. Because it is undeniable: one goes to hospital to get treatment, certainly not to be pampered in the first place. If he gets better, even if he got his ass kicked it's okay, you know. The point is when this does not happen, and in any case the aspect of listening is equally important, the aspect of finding a comfortable environment, of being listened to... [Interviewee n. 10]

#### End-of-life

A second thematic category concerns end-of-life issues. Within this broad category, several issues were reported by the interviewees.

Among the most recurrent ones is the implementation of advanced treatment provisions, commonly referred to as “living will”, and the advanced care planning, i.e., the statement patients can sign in anticipation of a clinical deterioration by advancing their preferences regarding treatment options (such as the request not to be resuscitated under certain conditions or not to undergo a tracheotomy if in respiratory failure). Some interviewees (n. 2, 7, 12, 14) reported that these arrangements, whilst provided by national law, are not currently enforced at the Institution. Patients are rarely asked explicitly for prior authorization to undergo or refuse certain medical treatments:

There is often no awareness of what the patient wanted in terms of critical area and perspectives of a life that is not completely autonomous. [Interviewee n. 2]

As a result, doctors are forced to adopt a conservative approach, even if they feel they are not acting in the best interests of their patients.

And we, I mean us doctors who are on call in the wards, we find ourselves in this dilemma especially on call nights (…). And it happens that you're suddenly called at night because a patient has been sick... We are called if a patient is sick. But, of course, we don't know all the patients of the Institution. So, before starting the shift, in some department doctors leave the instruction if, for instance, there is a patient who has given instructions not to be resuscitated or if the doctor himself, after talking to the patient, said that the situation is so advanced that it is not appropriate to subject the patient to procedures that would not be advantageous for them. This happens in very few if not in a single ward, this passing on of information. (…) I found myself there, that you have the emergency - life or death of the patient - and you don't have the chance to understand or know what the patient would want. [Interviewee n. 7]

In some cases, it is family members who explicitly request treatments that operators themselves perceive as disproportionate: some healthcare professionals report emotional and moral struggle to cope with relatives demanding to implement disproportionate therapies or continuing therapies that are more burdensome than beneficial to patients (n. 2–4, 7, 15, 16, 19).

#### Resource allocation

Another theme that emerged from many interviews is resource allocation (n. 1, 8–10, 13, 15). According to our participants, this issue is particularly relevant where resources are limited—e.g., in terms of medical time or availability of drugs, as in the case of experimental therapies—and clinicians often have to decide on their own how to allocate them among patients (n. 8–10). When patients present an uncertain prognosis or a rare disease, the lack of data or the vagueness of guidelines does not always allow for evidence-based decision-making.

(About patients with uncertain prognosis) What to propose, what to do, towards complicated situations, where there is a great level of uncertainty concerning the effectiveness of a treatment? A great level of uncertainty especially about the benefit that one choice or another can give to the patient, considering also the expense, the social expense, the physical expense for the patient, the cost... In short, in terms of a cost-benefit rationale to all intents and purposes, I mean. In other words, on the one hand, we have a doubtful benefit that we can give to a human life. On the other hand, we have all the other things: the risk of damaging the patient, the risk of worsening one' s quality of life, one' s survival, the economic damage that can be done to the National Healthcare System, or the social damage, or... [Interviewee n. 8]

Other interviewees also point to an unequal distribution of resources across the territory, with highly specialized centres located in some areas more than in others. This leads to long waiting lists and to a potential delay in diagnosing which may in turn have an impact on the starting of the treatment (n. 1, 5, 11, 15, 18). Also, some stakeholders underline the heterogeneous criteria for drug reimbursement across the country, which leads to potential economic disadvantages for patients (n. 1, 13, 15). Although these issues may appear far from the ambulatory reality, they actually pose questions also at the level of daily clinical practice:

This rather grey area is not ethically irrelevant. Because it poses the clinician the problem: I have in front of me a patient who could benefit from this drug, but he has to pay for it. Can he pay for it? [Interviewee n. 13]

### Interviewees’ preferences about clinical ethics support services

Questions from 7 to 10 (see Additional file 1: Appendix 1) aimed at exploring participants’ preferences on the topic of clinical ethics support services, with the aim of understanding how they currently manage clinical ethics issues and how they would like to be supported in the future, both individually and as units in the management of controversial clinical ethics issues.

First, we observed that most interviewees were unaware of the reality of CESS (19 out of 21 participants): they were not aware of all institutional forms of clinical ethics support systems and their functions. Among them are also 6 of the 8 REC members we interviewed, even though the current REC is theoretically required to provide this type of service as well.

When asked about the ideal role and desired activities of a CESS, almost all the participants [1, 5–17, 19, 20] replied that they would like more training in bioethics, and to have a space to discuss ethically complex clinical cases and the most common ethical issues in the institution.

In the next sections, we will report findings related to specific aspects of CESS that emerged during the interview.

#### How do healthcare professionals manage clinical ethics issues?

According to our sample, the Fondazione IRCCS Istituto Nazionale dei Tumori still lacks *ad hoc* protocols for the management of ethically concerning clinical issues (n. 3–6, 18), and the responsibility for conflict resolution falls entirely on the clinicians (n. 2, 3, 4, 14). As a result, healthcare professionals—physicians in particular—report that they deal with ethical issues in the same way as they do with clinically complex cases: they resort to discussion among colleagues of their own medical equip (n. 2–5, 8, 10, 13, 19). Some are used also to contact other specialists when needed, such as physicians from other wards or other health professionals, especially psychologists (55.5%) and, more rarely, the hospital chaplain (n. 3,4):

We are very used to sharing, meaning that in my opinion the worst thing that can happen when faced with a difficult decision is to make it alone. So, we see each other every day and the goal of the daily meeting is to meet to discuss difficult cases. We see each other once a week in a multidisciplinary context and in this context, we discuss multidisciplinary difficult cases. So, I would say that sharing is our (...) best weapon. [Interviewee n. 8]

Interviewees report that, in the most complex cases and if a solution cannot be shared between doctor and patient, it may be necessary to resort to the court (n. 3, 14). Or again, physicians may be forced to choose a downward compromise, which does not necessarily correspond to the patient’s best interests (n. 2, 7).

In general, clinicians appeal to interprofessional exchange and common sense:

(Such complex issues) basically are handled with much good will, with much good will, meaning that it is the human factor that makes things go in the right direction at the right time. However, this is not always possible: there are wards, there are working groups where this is lacking, for different reasons. (…) I mean, the solution is surely to have more resources. Surely to also have the possibility to, you know, to have networks of collaboration. [Interviewee n. 15]

#### What kind of clinical ethics support service would satisfy stakeholders’ preferences?

The questions concerning the preferences about CESS (questions 7–10) were aimed at identifying the thematic issues and purposes for which the interviewees would make use of a clinical ethics support service, if available. Based on these findings, we expected to identify the features of this service as well as the functions it should perform in order to meet the needs of the stakeholders working within the Fondazione IRCCS Istituto Nazionale dei Tumori.

Most interviewees expressed the desire to have a body, namely an ethics committee, to which report clinical ethical issues and ask for support and advice (interviewees n. 1, 3–5, 7–10 12, 14–16, 19).

So sometimes, especially for those of us who sometimes get somewhat involved to face delicate choices and then ... Sometimes you, so to say, you need an additional interlocutor, besides your professionalism, someone to discuss with... A moment of reflection, that's it, because that's what's missing. [Interviewee n. 5] (…) there are situations in which, as I told you at the beginning, it is only through sharing, discussion, confrontation... Here, however, we need the humility that us doctors very often do not have. It seems to us that our experience is everything; it's not like that, you know? And so, having also... I always believe in external help. [Interviewee n. 16]

Others wished to have a space to discuss general problems affecting clinical practice, such as access to innovative treatments for patients at a very advanced stage or hospital policy on second opinion requests (n. 13, 17). Some also pictured the support service as a body for supervising how healthcare professionals handle ethical issues in clinical practice; in this scenario, the committee’s members would have to do a sort of “ethical performance review”, analysing the staff’s management of complex cases (n. 16, 19).

I haven't had clinical activity for 15 years now, but that is a context in which what we were saying, supervision, would be vital, wouldn't it? A support, a sharing. Also, because in those situations you are so involved that it is absolutely necessary to have a context that allows you to be understood and, from a certain point of view, I would also say evaluated. [Interviewee n. 6]

For a few interviewees, the presence of a clinical ethics support service where problematic cases can be discussed represents an opportunity to be aware of what is the institutional viewpoint and to share the responsibility for the choice, considered either as legal liability (n. 13, 14) or as moral burden (n. 5, 8, 21), or both (n. 3, 4).

Five interviewees stress that a CESS, implemented in the form of CEC, should not only provide answers to specific clinical ethics criticalities, but also play a more proactive role (n. 17). It should discuss and analyse the most common and relevant clinical ethics issues recurrent in the Institution, and consequently develop proper (or reviewing already existent) guidelines (n. 5, 12, 13) and operational protocols (n. 14) to advise and inform operators. Similarly, some operators reported that they would find it useful to have some guidance on how to address the most frequent clinical ethical issues arising in clinical practice (n. 9) or even the less common but nonetheless relevant ones, e.g., the treatment of minors from Jehovah’s Witnesses families (n. 3, 4). In this respect, some stakeholders explicitly emphasised that the development (or revision) of guidelines concerning clinical ethics issues would have an educational role, as it would help to create an ethical culture in the long term: they feel the effort to examine the most relevant and recurrent clinical ethics issues in the Institution in an attempt to rethink their management promotes ethical reflection (n. 5) and can lead, over time, to the development of shared strategies among operators (n. 7). According to others, rethinking operative protocols through the help of a devoted clinical ethics support service could be an opportunity to improve patient’s management (n. 14, 15, 20).

Another activity that participants indicated as important for an ideal CESS is training (n. 1, 6, 7, 14, 16, 17 and 19). Educating health care professionals on ethically sensitive issues occurring in clinical practice means, for the interviewed, raising professionals’ awareness and improving their ethical sensitivity (n. 1, 6, 16), thus making them potentially better prepared in the management of complex cases, preventing further complications and reduce cases of litigation with patients (n. 14) or conflicts between colleagues (n. 7). In this respect, communication is the area in which stakeholders perceive there is the greatest training need: educating operators to improve their communication with patients is perceived as a key element both to ensure a better therapeutic process and to prevent conflicts (n. 1, 7, 11, 14).

Other support activities were mentioned as potentially helpful and somewhat desirable: the service, as a *super partes* body, could act as mediator among colleagues (n. 8) or with patients and relatives (n. 3, 4, 15) in case of conflict; also, the CESS could act as a guarantor that the Institution takes (active) care of patients, providing them better support both in the management of their treatment (n. 9) and in some particularly difficult moments of their disease, such as the communication of the diagnosis to the patient and any worsening of the prognosis (n. 11, 18).

#### What kind of clinical ethics support service would satisfy stakeholders’ preferences?

Question n. 9 aimed at exploring participants’ preferences over the institutional arrangement of an ideal CESS to be implemented in the Fondazione IRCCS Instituto dei Tumori Milano. Not all participants expressed well-defined preferences on this topic: some interviewees only declared themselves in favour of a committee (n. 9) or individual ethics consultant (n. 3, 4, 11, 18) arrangement. Two participants did not express any opinion on this matter (n. 15, 20). In general, most stakeholders believe that a CESS organized in the form of a CEC may be the most suited to the context of the Institution: this would indeed allow a multidisciplinary composition which may help promoting discussion among members (n. 2, 6–9, 12, 14, 19, 21):

If the culture of multidisciplinarity were to develop, a culture in which everyone really listens and is listened to (…) I think that, if in all these areas we talked about there was the space and time to confront each other, the issues would already be well managed and all. [Interviewee n. 5]

Also, a plurality of perspectives is thought to guarantee less subjective and/or partisan considerations (n. 7, 12, 21) and to help ensuring that the clinical ethics support service does not voice a single moral perspective (n. 7, 9, 12). Participants n.6 and n.13 explicitly state that the moral pluralism characterizing our societies should be represented within the committee and guaranteed by the presence of a plurality of members.

Beside the multidisciplinarity, some stakeholders believe that an ideal service should have a flexible structure, to better fit the different needs and circumstances that may arise (n. 1, 5, 8, 13, 16, 17). They would like a complex structure, variable upon activity: an entire committee, devoted to policy development and/or revision and discussion of non-urgent cases, and individual ethics/bioethics consultants or a small group of consultants, who can offer support in urgent cases that require immediate intervention (n. 1, 5, 8), provided that these consultant report to the main ethics committee after each consultation (n. 13, 16, 17).

#### Who should provide clinical ethics support?

Few participants expressed explicit preferences about the ideal composition of a potential clinical ethics support service. As already reported, multidisciplinarity is the most relevant feature for a fair decision-making process, according to our sample, as it requires the participation of professionals belonging to different fields and with complementary skills and experiences (n. 1–6, 12–14, 16). Only four interviewees identified professionals they consider essential among the ideal committee members (n. 1–4, 13): besides physicians, the most mentioned profile is the clinical ethics expert or bioethicist (n. 1–4, 13), followed by the psychologist (n. 1, 2), the expert in communication, the patient advocate, and the forensic doctor (n. 13).

## Discussion

### Preliminary considerations: difficulties in identifying ethical issues

The first important aspect that emerges from the interviews is a set of difficulties for healthcare personnel to identify and define an ethical problem and its implications. When asked to report a clinical ethics issue (or, in a simpler manner, a clinical issue presenting also an ethical/moral trait/layer), often clarifications and/or examples were asked for. Likewise, participants often reported issues that are at the cross-roads of ethical, psychological, and spiritual domains, along with problems of a completely different nature. This shows that, even though over one third of our sample stated that they had received at least some form of bioethics training, this may not be enough to cope with the significant challenges that clinical practice poses on an everyday basis. This may be due also to issues pertaining to training or otherwise to a lack of practice.

Indeed, most participants defined the problems they reported as “communication problems”. In this macro-area, they included a very multifaceted set of issues and themes that are not actually limited to communication. Although some communication problems may be ethically relevant and drawing a line is not always possible, our results indicate that a first step could be to help healthcare staff recognize the ethical aspects of the problems they face in their clinical daily practice.

However, through coding and analysis, some clinical ethical issues indeed emerged and we were able to categorize them, as shown in Table 2. Amongst them, we focus on the same issues presented in details in the Results section: namely, communication and informed consent, end-of-life, and resource allocation. In the following paragraphs, we also discuss the reported stakeholders' opinions and expectations concerning a potential ethics support service.

### Communication and informed consent process

As anticipated, the theme of communication was pointed out as one of the most relevant and problematic by interviewees. Within this broad theme, however, we felt the need to make a distinction between issues related to the content and the process of communication.

Regarding the former, we may observe that operators encounter significant difficulties in communicating a deterioration of the medical condition to the patient and its potential implications [36]. The news of a prognostic worsening and the nearing of death is not always straightforward or clear, and doctors may resort to periphrases or metaphors to avoid using words such as 'terminal' or 'death' [37]. Such communication issues may lead to consequences [38] in terms of self-determination capabilities, affecting patient’s autonomy in decision-making [39]. This is a well-known issue in oncology [40–43].

In regard to the process of communication, including a reported lack of empathy, it should be noted that how physicians communicate information may not be substantial in the care path, but it acquires different values depending both on the individual patient’s experience and on his/her prognosis [44]. If the lack of empathy may be tolerated and overlooked in the case of a full recovery, when facing a poor prognosis, these (seemingly secondary) aspects acquire greater importance: feeling understood and accompanied in the last phases of life makes a significant difference to the patients’ quality of life [45].

### End-of-life

The second theme is the end-of-life. End-of-life is linked to any medical context, but, in a more robust manner, to the oncology context, as it inevitably affects different aspects related to the treatment path.

As already said, one of the issues raised by participants concerned the lack of proper advance treatment provisions’ implementation within the institution. In Italy a law provides for this option [46], but this instrument is not yet fully implemented in real practice. It may then happen that patients at an advanced stage of illness become unconscious or in need of resuscitation, but they have not signed any disposition nor left their will in oral form, thus leaving the intensive care or on call doctors with no guidance on their therapeutic preferences. Problems arise when these patients have not had the opportunity to express their wishes, due to some lack of properly shared information about their prognosis, treatment options and/or their rights to refuse unwanted future medical interventions. The occurrence of such problems might be limited by specific interventions.

Linked to this issue is the matter of assisted suicide. Although it may not concern a large percentage of patients, this topic is particularly relevant today as a result of the sentence 242/19 of the Italian Constitutional Court [47]. In the absence of an *ad hoc* law on assisted suicide, the Constitutional Court recently decriminalized the aid to suicide under certain conditions and entrusts Ethics Committees with the responsibility for protecting vulnerability. Of course, the issue itself does not only concern Ethics Committees operating in cancer institutions, but all Italian Ethics Committees in general, which therefore will find themselves in the position to make decisions on a matter that would formally fall within their competence but which they rarely deal with in daily practice. As we have already mentioned, most Ethics Committees are overburdened by the ethical review of clinical trials and do not usually have the resources to deal with the ethical issues of clinical practice [13]. Furthermore, this issue is particularly relevant in oncology, since several studies have found a much higher suicide rate among cancer patients than the general population [48–50].

In conclusion, the issues related to withdrawing or withholding therapies in the terminal stages of a disease seem particularly relevant for stakeholders working with cancer [51–54]. In addition to the patient's clinical status, and the severity of prognosis, many different aspects are involved, such as the religion and culture of the individual patient, but also the doctor's attitude towards end-of-life and the regulatory framework in which he or she operates [55]. Complex concepts of great ethical importance, such as the respect for patient's autonomy and values, quality of life, and the notion of proportionality of treatment, also play an important role. In conclusion, it is hard to imagine that doctors without full ethical awareness and a strong ability to analyse ethical problems would be comfortable dealing with such issues on their own.

### Resource allocation

The last theme that we discuss is related to some decisions in clinical practice which in some way have to do with allocation of medical resources. This very traditional issue within bioethics literature^2^ was referred to by some stakeholders. These decisions may be challenging, as they are often related to conditions in which either evidence is scarce, or the limitation of resources is subtle.

Deciding how to deliver a limited resource, even when the limitation is not so straightforward and mainly results into waiting lists and the like, represents a paradigmatic problem of multidisciplinary expertise, with the potential of ethical implications which may be hard to make explicit.

If we consider the specific (national) context of this investigation, as a matter of fact there may be differences in the distribution of some healthcare resources, for example on rare cancers; this may represent a source of potential discrepancies in the actual access of patients to available facilities. Despite the national networks established over the decades (e.g., the Italian Rare Cancer Network), a degree of health care migration does exist as a matter of fact. On another note, a problem may be the time interval to reimbursement of drugs approved by the European Medicines Agency (EMA): the Italian Medicine Agency (AIFA) statistically takes on average up to one year (352 days) to complete the pricing negotiation procedure for a drug [56]. Thus, there may be the case of a drug approved by the EU regulator and hence purchasable, but not yet reimbursable by the Italian health system. Furthermore, once AIFA decides upon a drug redeemability, each Region can establish further restrictions on the reimbursement handbook, resulting in the same drug being reimbursable in one Region and not in another. Traveling for health reasons is of course possible, but it represents a cost and can therefore be a significant economic strain for some segments of the population [57].

### Preferences about clinical ethics support services

Concerning the potential implementation of a CESS, stakeholders would like to have an independent body to which they could address doubts and questions on ethically complex clinical cases and wish they could rely on more in-depth guidelines or shared strategies at institutional level on issues that occur frequently in the hospital. Many perceive a need for additional training in bioethics and clinical ethics among healthcare staff. Although expressed in lay and non-technical terms, these tasks cover at least partially the standard functions that the literature attributes to CECs: namely, ethics consultation, bioethics training, and drafting and/or revising policy and guidelines.

Despite some differences in its ideal characteristics and in line with literature [18], clinical ethics consultation, interpreted as case discussion at the least, emerged as the most desired activity.

### Strategies to address clinical ethics issues

Stakeholders’ responses on this issue show that there is a lack of institutional facilities devoted to the management of ethical issues. Healthcare staff often relies on multidisciplinary meetings, where different specialists discuss the most complex cases encountered in their practice [58–61]. For some interviewees, such multidisciplinary meetings also represent an opportunity to share their decisional responsibility with other members of the team, benefiting from other experts’ perspectives.

Meetings among medical professionals seem to be the main available institutional strategy to deal with the most complex cases. However, it should be noted that such meetings generally involve only physicians, though from different medical areas: oncologists, surgeons, radiotherapists, radiologists, anatomopathologist. Other important categories of health professionals, such as nurses and psychologists may not be present or may be only occasionally consulted. In the context of ethics committees, this would imply, for instance, the involvement of professionals belonging to different fields, not exclusively medical [62].

From what emerged from the interviews, it seems that the management of the most ethically complex issues is often left to the common sense and good will of single individuals, that would be the only guiding criteria in the absence of specific dedicated guidelines or other institutional support [18, 63]. This means that a key part of care is left to be handled by individual clinicians on the basis of their own personal skills and ethical awareness.

Although medical culture is moving increasingly in the direction of personalized medicine [64–67], treatment paths seem to be still organized considering only the clinical needs of patients. Little attention is paid to support for health care professionals when dealing with ethically sensitive clinical cases [13].

As a result, health care professionals are left alone to decide on these issues and bear full responsibility for them. Thus, the proper management of ethically relevant issues is delegated to individual and subjective criteria of physicians who may have inadequate training in ethics and thus may not be fit to effectively deal with ethical issues.

As reported above, multidisciplinary confrontation is experienced as an aid in the management of the decision burden and therefore as a sharing of responsibility. However, this may not be enough, and the possibility to rely on a clinical ethics support service seems desirable for all the participants in the study, with no exception.

However, they express different—and sometimes conflicting—wishes about the ideal functioning of such a service. If some interviewees imagine a service that acts primarily on need, and thus on individual cases, in a way comparable to what the literature refers to as the activity of clinical ethics consultation, the first step to raise awareness on ethical issues is training, in line with most interviewees’ opinion.

In any case, from all participants interviewed it emerged the desire, if not the need, to have a space dedicated to reflect over the ethics of medical care and clinical decision making, where healthcare professionals and operators can report the problems they face in clinical practice and have feedback and/or guidance from experts on the subject.

### Limitations

This study presents the same shortcomings of other qualitative works.

First, the findings reported and discussed come from a limited number of participants and some relevant clinical areas remain unexplored, since we did not receive a reply from all operational units.

Moreover, since participation in the study was on a voluntary basis and required a minimum time of 30 min, a selection bias in our sample cannot be excluded: it is likely that only or mainly professionals who are already sensitive to ethical-clinical issues have made themselves available.

Another possible limitation concerns the fact that some interviewees are members of the Institution’s REC. Their membership may have influenced our results. Indeed, on one hand, Ethics Committee’s members have undeniable expertise in ethics and are used to reasoning over bioethical issues. On the other hand, however, the issues they deal with concern mainly research.

## Conclusions

The results of our study confirmed the well-established finding in literature that healthcare professionals are often facing ethically relevant clinical issues at the patient bedside. The critical context of end-of-life care appears to be a major source of ethical issues in oncology, as well as and perhaps more than in other clinical areas. Subtle matters of resource allocation in real-world clinical practice, however, may be more prevalent, or more appreciated, in oncology centres than in other general hospitals.

However, most of the staff members among the participants in our study stated that they lack expertise and theoretical knowledge in clinical ethics. Even those who are involved in research ethics, such as the members of the REC, may not be necessarily competent in clinical ethics as well. On the other side, there is a lack of institutional facilities devoted to the resolution of clinical ethics issues. As a result, healthcare personnel are often left alone in dealing with such issues, mostly relying on multidisciplinary clinical discussions as an opportunity to feel less overwhelmed.

The positive and encouraging feedback we received with respect to the purpose of our study clearly indicates an interest towards these issues. Likewise, the replies of the enrolled participants confirm the desire of healthcare professionals to have a space where they can discuss their ethical concerns and receive support as needed.

What observed in this study at an important Italian comprehensive cancer centre is likely similar to what experienced at many other cancer centres. If referred to the Italian context, our data confirm the need, repeatedly pointed out by the relevant bodies such the INCB [16] and by international bioethics literature [68–70], to integrate the therapeutic pathway with an ethical dimension and to speed up the development of CECs [10].

If we consider the specificity of the oncological context, an increased sensitivity for the ethical dimension of clinical care and its humanization becomes even more relevant: addressing the issues related to unclear and non-transparent communication and end-of-life care is crucial for patients suffering from a life-threatening and high-impact disease such as cancer.

## Supplementary Information

**Additional file 1: Appendix.** List of draft questions that guided each semi-structured in-depth interview.** Related file 1. ISSM COREQ Checklist**—Ethical issues in oncology practice. This file contains the COREQ checklist for qualitative research by Tong et al. (2007), used to both develop the research design and methodology of the present manuscript and to report data in the Methods and Results section. All the page references for the relevant items have been reported in the checklist.

## Data Availability

The datasets generated and analysed during the current study are not publicly available due to confidentiality and privacy reasons: the transcripts of the interviews contain information from which the identity of the participant can be easily retraced, thus we could include in the manuscript only excerpts of the raw material to support the findings and conclusions, along with the outline of the interview questions. However, the datasets are available from the corresponding author on reasonable request.

## Abbreviations

CESS: Clinical Ethics Support Services
CEC: Clinical Ethics Committee
REC: Research Ethics Committee
INCB: Italian National Committee for Bioethics
AIFA: Italian Medicine Agency
EMA: European Medicines Agency

## Footnotes

1 Of the REC members, one belongs also to a Patient Advocacy group, while another is also a nurse.

2 We may recall, amongst others, the case related to God’s Committee and kidney patients, which contributed to the birth and diffusion of clinical ethical committees, which was a problem of allocation of scarce resources (69–71).
