# Supplementary material for: Ethical issues in oncology practice: a qualitative study of stakeholders’ experiences and expectations
Source: BMC Med Ethics. 2022 Jun 30;23:67. doi: 10.1186/s12910-022-00803-x (PMC9248199; doi:10.1186/s12910-022-00803-x)
Supplement: Supplementary file 1 — Additional file 1: Appendix. List of draft questions that guided each semi-structured in-depth interview. Related file 1. ISSM COREQ Checklist—Ethical issues in oncology practice. This file contains the COREQ checklist for qualitative research by Tong et al. (2007), used to both develop the research design and methodology of the present manuscript and to report data in the Methods and Results section. All the page references for the relevant items have been reported in the checklist. [file 12910_2022_803_MOESM1_ESM.docx]

# **Appendix 1**

1. In your activity as a doctor/nurse, did you face any clinical case with raising ethical issues? Which ones? Can you give me a few examples?

2. In your experience, which are the most common ethical issues and which would require immediate action?

3. Do you feel that patients are experiencing the same issues? Or do you think that patients point out other issues?

4. What was the most difficult ethical experience you had to deal with, from an ethics perspective? Why? How did you manage it?

5. Did you need external support? Yes/No, from whom?

6. Do you occasionally discuss ethical issues with other staff members?

7. What kind of ethical support service do health professionals need?

8. In your opinion, does a doctor need the same kind of support as other health professionals? If yes, why? If not, why?

9. In your opinion, what kind of body would be best suited to provide ethics support in the Institution? A single expert/a committee?

10. What should this body deal with in order of priority?
